# 2,5-Dimethyl-1-phenyl­sulfonyl-1*H*-pyrrole-3,4-dicarbaldehyde

**DOI:** 10.1107/S1600536809004425

**Published:** 2009-02-18

**Authors:** P. R. Seshadri, B. Balakrishnan, K. Ilangovan, R. Sureshbabu, A. K. Mohanakrishnan

**Affiliations:** a Postgraduate and Research Department of Physics, Agurchand Manmull Jain College, Chennai 600 114, India; b Department of Physics, P. T. Lee Chengalvaraya Naicker College of Engineering and Technology, Kancheepuram 631 502, India; c Postgraduate and Research Department of Physics, RKM Vivekananda College, Chennai 600 004, India; d Department of Organic Chemistry, University of Madras, Guindy Campus, Chennai 600 025, India

## Abstract

In the title compound, C_14_H_13_NO_4_S, the mean planes of the pyrrole and phenyl rings form a dihedral angle of 88.7 (1)°. The aldehyde groups are slightly twisted from the pyrrole plane. In the crystal structure, mol­ecules are linked into a three-dimensional framework by C—H⋯O hydrogen bonds.

## Related literature

For general background, see: Ali *et al.* (1989 ▶); Amal Raj *et al.* (2003 ▶). For bond-length data, see: Allen *et al.* (1987 ▶). For N-atom hybridization details, see: Beddoes *et al.* (1986 ▶).
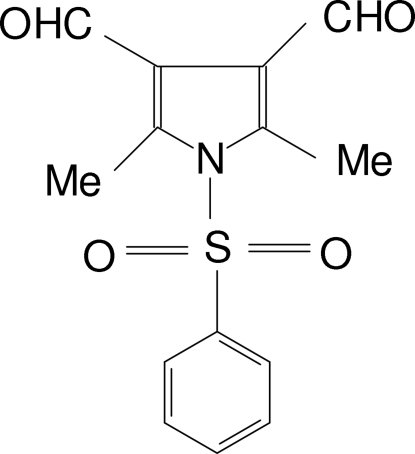


## Experimental

### 

#### Crystal data

C_14_H_13_NO_4_S
*M* *_r_* = 291.31
Monoclinic, 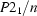

*a* = 9.0257 (3) Å
*b* = 12.6240 (5) Å
*c* = 11.9914 (5) Å
β = 97.700 (2)°
*V* = 1353.99 (9) Å^3^
*Z* = 4
Mo *K*α radiation
μ = 0.25 mm^−1^
*T* = 293 K
0.25 × 0.20 × 0.20 mm

#### Data collection

Bruker Kappa APEXII area-detector diffractometer
Absorption correction: multi-scan (*SADABS*; Sheldrick, 2001 ▶) *T*_min_ = 0.940, *T*_max_ = 0.952
19609 measured reflections
4736 independent reflections
3164 reflections with *I* > 2σ(*I*)
*R*_int_ = 0.032

#### Refinement

*R*[*F* ^2^ > 2σ(*F* ^2^)] = 0.048
*wR*(*F* ^2^) = 0.154
*S* = 1.00
4736 reflections
183 parameters
H-atom parameters constrained
Δρ_max_ = 0.19 e Å^−3^
Δρ_min_ = −0.42 e Å^−3^

### 

Data collection: *APEX2* (Bruker, 2004 ▶); cell refinement: *SAINT* (Bruker, 2004 ▶); data reduction: *SAINT*; program(s) used to solve structure: *SHELXS97* (Sheldrick, 2008 ▶); program(s) used to refine structure: *SHELXL97* (Sheldrick, 2008 ▶); molecular graphics: *ORTEP-3* (Farrugia, 1997 ▶) and *PLATON* (Spek, 2009 ▶); software used to prepare material for publication: *PARST* (Nardelli, 1995 ▶).

## Supplementary Material

Crystal structure: contains datablocks I, global. DOI: 10.1107/S1600536809004425/wn2307sup1.cif

Structure factors: contains datablocks I. DOI: 10.1107/S1600536809004425/wn2307Isup2.hkl

Additional supplementary materials:  crystallographic information; 3D view; checkCIF report

## Figures and Tables

**Table 1:** Hydrogen-bond geometry (Å, °)

*D*—H⋯*A*	*D*—H	H⋯*A*	*D*⋯*A*	*D*—H⋯*A*
C6—H6⋯O3	0.93	2.46	3.077 (2)	124
C7—H7*A*⋯O4	0.96	2.33	3.021 (3)	128
C11—H11⋯O4^i^	0.93	2.53	3.456 (2)	174
C13—H13⋯O2	0.93	2.52	3.304 (2)	143
C14—H14⋯O3^ii^	0.93	2.57	3.383 (2)	146

Symmetry codes: (i) 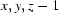; (ii) 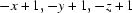.

**Fig. 1.**
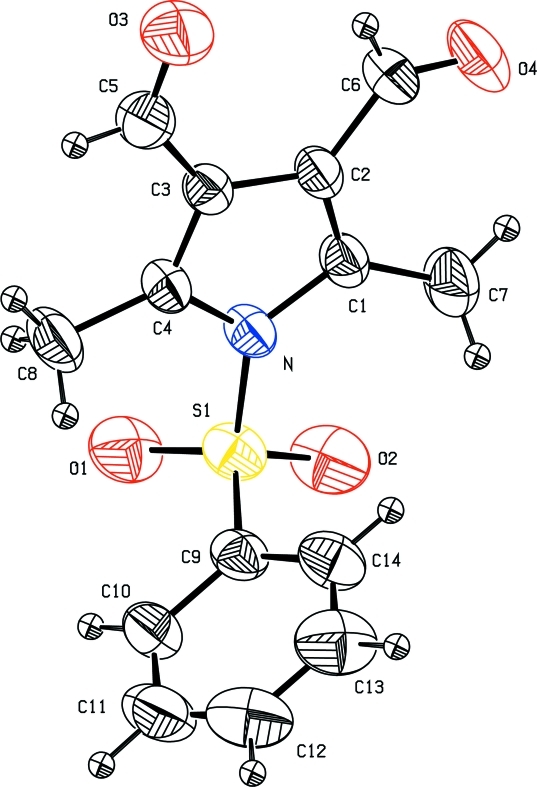
Molecular structure of the title compound showing 30% probability displacement ellipsoids. Hydrogen atoms are represented by spheres of arbitrary radius.

**Fig. 2.**
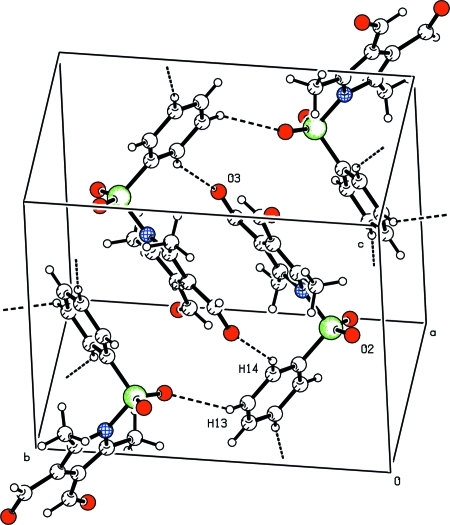
Crystal packing of the title compound. Hydrogen bonds are shown as dashed lines.

## supplementary crystallographic information

### Comment

Heterocyclic compounds, especially five-membered rings, have occupied an important place among organic compounds because of their biological activities. The fungicidal activity of novel heterocycles has been reported by Ali *et al.* (1989). These are crucial intermediates for various pyrrole natural products possessing antitumour properties. They are found to have antifungal activity against various pathogens (Amal Raj *et al.*, 2003). Against this background and in order to obtain detailed information on molecular conformation in the solid state, an X-ray crystallographic study of the title compound has been carried out and the results are presented here.

The geometric parameters are normal (Allen *et al.*, 1987). The mean planes of the pyrrole and phenyl rings form a dihedral angle of 88.7 (1)°. The aldehyde groups are slightly twisted from the pyrrole plane, with O3 towards C2 and O4 towards C1, as evidenced  by the torsion angles C2—C3—C5—O3 = 2.1 (3)° and C1—C2—C6—O4 = 5.9 (3)° (Fig. 1). The sum of the angles at N is 360.0, which is an indication of *sp*^2^ hybridization (Beddoes *et al.*, 1986). In the crystal structure, the molecules are linked into a three-dimensional framework by C—H···O hydrogen bonds (Fig. 2 and Table 1).

### Experimental

To a suspension of 3°-butoxide (4.4 g, 39.7 mmol) in dry THF (20 ml), 18-crown-6 (catalyst) and 2,5-dimethyl-1*H*-pyrrole-3,4-dicarbaldehyde (4 g, 26.5 mmol) in dry THF were added slowly at room temperature and the reaction mixture was stirred for 15 min. To this, PhSO_2_Cl (6.1 g, 34.4 mmol) in dry THF (15 ml) was added and stirred for another 4 h. The mixture was then poured over ice–water (500 ml) and the solid obtained was filtered. The product, 2,5-dimethyl-1-(phenyl sulfonyl)-1*H*-pyrrole-3,4-dicarbaldehyde, was recrystallized from methanol. Yield 4.9 g (64%). M.p = 395 K.

### Refinement

All H atoms were positioned geometrically and allowed to ride on their parent atoms, with C—H = 0.93–0.96 Å and U_iso_(H) = 1.5 *U*_eq_(C) for methyl H atoms and 1.2*U*_eq_(C) for other H atoms.

### Crystal data

**Table:**

C_14_H_13_NO_4_S	*F*(000) = 608
*M**_r_* = 291.31	*D*_x_ = 1.429 Mg m^−^^3^
Monoclinic, *P*2_1_/*n*	Mo *K*α radiation, λ = 0.71073 Å
*a* = 9.0257 (3) Å	Cell parameters from 6814 reflections
*b* = 12.6240 (5) Å	θ = 2.4–32.2°
*c* = 11.9914 (5) Å	µ = 0.25 mm^−^^1^
β = 97.700 (2)°	*T* = 293 K
*V* = 1353.99 (9) Å^3^	Block, colourless
*Z* = 4	0.25 × 0.20 × 0.20 mm

### Data collection

**Table:**

Bruker Kappa-APEXII area-detector diffractometer	4736 independent reflections
Radiation source: fine-focus sealed tube	3164 reflections with *I* > 2σ(*I*)
graphite	*R*_int_ = 0.032
ω scans	θ_max_ = 32.2°, θ_min_ = 2.4°
Absorption correction: multi-scan (*SADABS*; Sheldrick, 2001)	*h* = −13→10
*T*_min_ = 0.940, *T*_max_ = 0.952	*k* = −18→18
19609 measured reflections	*l* = −16→17

### Refinement

**Table:**

Refinement on *F*^2^	Primary atom site location: structure-invariant direct methods
Least-squares matrix: full	Secondary atom site location: difference Fourier map
*R*[*F*^2^ > 2σ(*F*^2^)] = 0.048	Hydrogen site location: inferred from neighbouring sites
*wR*(*F*^2^) = 0.154	H-atom parameters constrained
*S* = 1.00	*w* = 1/[σ^2^(*F*_o_^2^) + (0.0787*P*)^2^ + 0.2246*P*] where *P* = (*F*_o_^2^ + 2*F*_c_^2^)/3
4736 reflections	(Δ/σ)_max_ < 0.001
183 parameters	Δρ_max_ = 0.19 e Å^−^^3^
0 restraints	Δρ_min_ = −0.42 e Å^−^^3^

### Special details

**Table:**

Geometry. All e.s.d.'s (except the e.s.d. in the dihedral angle between two l.s. planes) are estimated using the full covariance matrix. The cell e.s.d.'s are taken into account individually in the estimation of e.s.d.'s in distances, angles and torsion angles; correlations between e.s.d.'s in cell parameters are only used when they are defined by crystal symmetry. An approximate (isotropic) treatment of cell e.s.d.'s is used for estimating e.s.d.'s involving l.s. planes.
Refinement. Refinement of *F*^2^ against ALL reflections. The weighted *R*-factor *wR* and goodness of fit *S* are based on *F*^2^, conventional *R*-factors *R* are based on *F*, with *F* set to zero for negative *F*^2^. The threshold expression of *F*^2^ > σ(*F*^2^) is used only for calculating *R*-factors(gt) *etc*. and is not relevant to the choice of reflections for refinement. *R*-factors based on *F*^2^ are statistically about twice as large as those based on *F*, and *R*- factors based on ALL data will be even larger.

### Fractional atomic coordinates and isotropic or equivalent isotropic displacement parameters (Å^2^)

**Table:**

	*x*	*y*	*z*	*U*_iso_*/*U*_eq_	
S1	0.51190 (5)	0.21083 (3)	0.25629 (3)	0.05026 (14)	
N	0.56878 (14)	0.29263 (9)	0.36572 (10)	0.0417 (3)	
O1	0.64275 (15)	0.17150 (11)	0.21659 (11)	0.0704 (4)	
O3	0.82639 (16)	0.54763 (12)	0.58440 (12)	0.0777 (4)	
O2	0.40939 (17)	0.13956 (10)	0.29555 (12)	0.0743 (4)	
O4	0.49857 (19)	0.36086 (17)	0.70677 (12)	0.1023 (6)	
C1	0.51247 (15)	0.29778 (11)	0.46937 (11)	0.0422 (3)	
C2	0.59280 (15)	0.37280 (11)	0.53213 (11)	0.0404 (3)	
C3	0.69918 (14)	0.41747 (11)	0.46621 (11)	0.0383 (3)	
C4	0.68371 (15)	0.36717 (11)	0.36473 (11)	0.0412 (3)	
C5	0.80621 (18)	0.50177 (13)	0.49571 (14)	0.0524 (4)	
H5	0.8646	0.5223	0.4412	0.063*	
C6	0.57651 (19)	0.40278 (16)	0.64774 (13)	0.0579 (4)	
H6	0.6327	0.4602	0.6779	0.069*	
C7	0.3863 (2)	0.23246 (17)	0.50008 (16)	0.0661 (5)	
H7A	0.3587	0.2571	0.5702	0.099*	
H7B	0.4168	0.1597	0.5075	0.099*	
H7C	0.3022	0.2386	0.4423	0.099*	
C8	0.7684 (2)	0.38292 (17)	0.26769 (14)	0.0662 (5)	
H8A	0.8349	0.4421	0.2825	0.099*	
H8B	0.6997	0.3965	0.2009	0.099*	
H8C	0.8253	0.3203	0.2571	0.099*	
C9	0.41822 (17)	0.29562 (12)	0.15557 (12)	0.0455 (3)	
C10	0.4612 (2)	0.29939 (16)	0.04944 (14)	0.0604 (4)	
H10	0.5403	0.2583	0.0315	0.072*	
C11	0.3837 (3)	0.3659 (2)	−0.02973 (16)	0.0767 (6)	
H11	0.4104	0.3696	−0.1019	0.092*	
C12	0.2683 (3)	0.42608 (17)	−0.00229 (17)	0.0780 (6)	
H12	0.2176	0.4707	−0.0561	0.094*	
C13	0.2260 (3)	0.42188 (16)	0.10310 (18)	0.0744 (6)	
H13	0.1476	0.4638	0.1207	0.089*	
C14	0.3000 (2)	0.35534 (13)	0.18281 (14)	0.0579 (4)	
H14	0.2708	0.3507	0.2541	0.069*	

### Atomic displacement parameters (Å^2^)

**Table:**

	*U*^11^	*U*^22^	*U*^33^	*U*^12^	*U*^13^	*U*^23^
S1	0.0664 (3)	0.0385 (2)	0.0462 (2)	−0.00334 (15)	0.00855 (17)	−0.00739 (14)
N	0.0513 (6)	0.0408 (6)	0.0347 (5)	−0.0058 (5)	0.0120 (5)	−0.0013 (4)
O1	0.0800 (8)	0.0655 (8)	0.0655 (8)	0.0209 (7)	0.0084 (6)	−0.0213 (6)
O3	0.0858 (9)	0.0823 (10)	0.0652 (8)	−0.0240 (7)	0.0105 (7)	−0.0272 (7)
O2	0.1039 (10)	0.0500 (7)	0.0681 (8)	−0.0305 (7)	0.0080 (7)	0.0000 (6)
O4	0.1016 (12)	0.1637 (17)	0.0496 (8)	−0.0353 (11)	0.0398 (8)	−0.0136 (9)
C1	0.0459 (7)	0.0463 (7)	0.0362 (6)	−0.0003 (5)	0.0121 (5)	0.0061 (5)
C2	0.0429 (6)	0.0467 (7)	0.0333 (6)	0.0049 (5)	0.0115 (5)	0.0032 (5)
C3	0.0418 (6)	0.0389 (7)	0.0347 (6)	0.0024 (5)	0.0074 (5)	0.0024 (5)
C4	0.0465 (7)	0.0430 (7)	0.0358 (6)	−0.0019 (5)	0.0123 (5)	0.0015 (5)
C5	0.0560 (8)	0.0535 (9)	0.0480 (8)	−0.0081 (7)	0.0078 (6)	−0.0012 (7)
C6	0.0595 (9)	0.0796 (12)	0.0366 (7)	0.0025 (8)	0.0143 (6)	−0.0055 (7)
C7	0.0662 (10)	0.0772 (12)	0.0595 (10)	−0.0208 (9)	0.0248 (8)	0.0053 (9)
C8	0.0784 (12)	0.0817 (13)	0.0445 (9)	−0.0255 (10)	0.0295 (8)	−0.0068 (8)
C9	0.0551 (8)	0.0435 (7)	0.0379 (7)	−0.0089 (6)	0.0067 (6)	−0.0098 (5)
C10	0.0643 (10)	0.0774 (12)	0.0411 (8)	−0.0095 (8)	0.0134 (7)	−0.0091 (8)
C11	0.0942 (15)	0.0952 (16)	0.0402 (9)	−0.0202 (12)	0.0072 (9)	0.0005 (9)
C12	0.1054 (16)	0.0665 (12)	0.0567 (11)	−0.0007 (11)	−0.0086 (11)	0.0022 (9)
C13	0.0900 (14)	0.0586 (11)	0.0710 (12)	0.0162 (10)	−0.0029 (10)	−0.0134 (9)
C14	0.0742 (11)	0.0527 (9)	0.0472 (9)	0.0036 (8)	0.0099 (8)	−0.0136 (7)

### Geometric parameters (Å, °)

**Table:**

S1—O2	1.4155 (13)	C7—H7A	0.9600
S1—O1	1.4209 (13)	C7—H7B	0.9600
S1—N	1.6945 (12)	C7—H7C	0.9600
S1—C9	1.7452 (17)	C8—H8A	0.9600
N—C4	1.4018 (17)	C8—H8B	0.9600
N—C1	1.4060 (17)	C8—H8C	0.9600
O3—C5	1.203 (2)	C9—C10	1.380 (2)
O4—C6	1.187 (2)	C9—C14	1.381 (2)
C1—C2	1.358 (2)	C10—C11	1.385 (3)
C1—C7	1.492 (2)	C10—H10	0.9300
C2—C3	1.4382 (18)	C11—C12	1.364 (3)
C2—C6	1.463 (2)	C11—H11	0.9300
C3—C4	1.3631 (19)	C12—C13	1.370 (3)
C3—C5	1.449 (2)	C12—H12	0.9300
C4—C8	1.4890 (19)	C13—C14	1.376 (3)
C5—H5	0.9300	C13—H13	0.9300
C6—H6	0.9300	C14—H14	0.9300
			
O2—S1—O1	119.94 (9)	H7A—C7—H7B	109.5
O2—S1—N	105.88 (7)	C1—C7—H7C	109.5
O1—S1—N	107.05 (7)	H7A—C7—H7C	109.5
O2—S1—C9	110.02 (8)	H7B—C7—H7C	109.5
O1—S1—C9	109.26 (8)	C4—C8—H8A	109.5
N—S1—C9	103.33 (7)	C4—C8—H8B	109.5
C4—N—C1	109.39 (11)	H8A—C8—H8B	109.5
C4—N—S1	123.39 (9)	C4—C8—H8C	109.5
C1—N—S1	127.22 (10)	H8A—C8—H8C	109.5
C2—C1—N	107.03 (11)	H8B—C8—H8C	109.5
C2—C1—C7	128.14 (13)	C10—C9—C14	121.40 (16)
N—C1—C7	124.83 (14)	C10—C9—S1	119.29 (14)
C1—C2—C3	108.37 (12)	C14—C9—S1	119.29 (12)
C1—C2—C6	126.27 (13)	C9—C10—C11	118.31 (18)
C3—C2—C6	125.35 (14)	C9—C10—H10	120.8
C4—C3—C2	108.18 (12)	C11—C10—H10	120.8
C4—C3—C5	123.06 (13)	C12—C11—C10	120.27 (18)
C2—C3—C5	128.76 (13)	C12—C11—H11	119.9
C3—C4—N	107.02 (11)	C10—C11—H11	119.9
C3—C4—C8	129.31 (14)	C11—C12—C13	121.2 (2)
N—C4—C8	123.67 (13)	C11—C12—H12	119.4
O3—C5—C3	125.90 (15)	C13—C12—H12	119.4
O3—C5—H5	117.1	C12—C13—C14	119.66 (19)
C3—C5—H5	117.1	C12—C13—H13	120.2
O4—C6—C2	126.33 (18)	C14—C13—H13	120.2
O4—C6—H6	116.8	C13—C14—C9	119.18 (16)
C2—C6—H6	116.8	C13—C14—H14	120.4
C1—C7—H7A	109.5	C9—C14—H14	120.4
C1—C7—H7B	109.5		
			
O2—S1—N—C4	172.55 (12)	C1—N—C4—C3	0.29 (16)
O1—S1—N—C4	43.51 (14)	S1—N—C4—C3	−179.53 (10)
C9—S1—N—C4	−71.78 (13)	C1—N—C4—C8	179.88 (15)
O2—S1—N—C1	−7.23 (15)	S1—N—C4—C8	0.1 (2)
O1—S1—N—C1	−136.28 (13)	C4—C3—C5—O3	−178.68 (17)
C9—S1—N—C1	108.43 (13)	C2—C3—C5—O3	2.1 (3)
C4—N—C1—C2	−1.03 (16)	C1—C2—C6—O4	5.9 (3)
S1—N—C1—C2	178.78 (10)	C3—C2—C6—O4	−173.12 (19)
C4—N—C1—C7	178.16 (15)	O2—S1—C9—C10	−122.33 (14)
S1—N—C1—C7	−2.0 (2)	O1—S1—C9—C10	11.30 (15)
N—C1—C2—C3	1.33 (16)	N—S1—C9—C10	125.00 (13)
C7—C1—C2—C3	−177.83 (16)	O2—S1—C9—C14	55.92 (14)
N—C1—C2—C6	−177.86 (14)	O1—S1—C9—C14	−170.46 (12)
C7—C1—C2—C6	3.0 (3)	N—S1—C9—C14	−56.75 (13)
C1—C2—C3—C4	−1.18 (16)	C14—C9—C10—C11	0.7 (3)
C6—C2—C3—C4	178.02 (14)	S1—C9—C10—C11	178.93 (14)
C1—C2—C3—C5	178.14 (14)	C9—C10—C11—C12	0.3 (3)
C6—C2—C3—C5	−2.7 (2)	C10—C11—C12—C13	−0.4 (3)
C2—C3—C4—N	0.53 (15)	C11—C12—C13—C14	−0.5 (3)
C5—C3—C4—N	−178.84 (13)	C12—C13—C14—C9	1.4 (3)
C2—C3—C4—C8	−179.04 (17)	C10—C9—C14—C13	−1.6 (2)
C5—C3—C4—C8	1.6 (3)	S1—C9—C14—C13	−179.77 (14)

### Hydrogen-bond geometry (Å, °)

**Table:**

*D*—H···*A*	*D*—H	H···*A*	*D*···*A*	*D*—H···*A*
C6—H6···O3	0.93	2.46	3.077 (2)	124
C7—H7A···O4	0.96	2.33	3.021 (3)	128
C11—H11···O4^i^	0.93	2.53	3.456 (2)	174
C13—H13···O2i	0.93	2.52	3.304 (2)	143
C14—H14···O3^ii^	0.93	2.57	3.383 (2)	146

Symmetry codes: (i) *x*, *y*, *z*−1; i; (ii) −*x*+1, −*y*+1, −*z*+1.
